# Saliva-based detection of SARS-CoV-2: a bibliometric analysis of global research

**DOI:** 10.1007/s11010-023-04760-w

**Published:** 2023-05-13

**Authors:** Chun Zhou, Zhaopin Cai, Boxing Jin, Huisong Lin, Lingling Xu, Zhigang Jin

**Affiliations:** 1 Jinhua People’s Hospital Joint Center for Biomedical Research, Zhejiang Normal University, Jinhua, 321000 Zhejiang China; 2 Department of Science and Education, the Affiliated Jinhua Hospital of Wenzhou Medical University, Jinhua, 321000 Zhejiang China; 3 College of Life Sciences, Zhejiang Normal University, 688 Yingbin Road, Jinhua, 321000 Zhejiang China; 4 Zhejiang Institute of Medical Device Testing, Hangzhou, Zhejiang China

**Keywords:** Saliva, SARS-CoV-2, COVID-19, Detection, CiteSpace, Bibliometric analysis

## Abstract

Saliva has emerged as a promising noninvasive biofluid for the diagnosis of oral and systemic diseases, including viral infections. During the coronavirus disease 2019 (COVID-19) pandemic, a growing number of studies focused on saliva-based detection of severe acute respiratory syndrome coronavirus-2 (SARS-CoV-2). Taking advantage of the WoS core collection (WoSCC) and CiteSpace, we retrieved 1021 articles related to saliva-based detection of SARS-CoV-2 and conducted a comprehensive bibliometric analysis. We analyzed countries, institutions, authors, cited authors, and cited journals to summarize their contribution and influence and analyzed keywords to explore research hotspots and trends. From 2020 to 2021, research focused on viral transmission via saliva and verification of saliva as a reliable specimen, whereas from 2021 to the present, the focus of research has switched to saliva-based biosensors for SARS-CoV-2 detection. By far, saliva has been verified as a reliable specimen for SARS-CoV-2 detection, although a standardized procedure for saliva sampling and processing is needed. Studies on saliva-based detection of SARS-CoV-2 will promote the development of saliva-based diagnostics and biosensors for viral detection. Collectively, our findings could provide valuable information to help scientists perceive the basic knowledge landscapes on saliva-based detection of SARS-CoV-2, the past and current research hotspots, and future opportunities.

**Supplementary Information:**

The online version contains supplementary material available at 10.1007/s11010-023-04760-w.

## Introduction

Since the outbreak of coronavirus disease 2019 (COVID-19), the detection of severe acute respiratory syndrome coronavirus-2 (SARS-CoV-2), the causative agent of COVID-19, has become central to effectively controlling the COVID-19 outbreak and has attracted intensive attention. The current gold standard for the detection of SARS-CoV-2 is the amplification of viral genome RNAs encoding structural proteins (S, N, M, and E proteins) or polyprotein orf1ab in nasopharyngeal or oropharyngeal specimens via real-time quantitative PCR (RT‒qPCR) [1, 2]. However, the collection of both nasopharyngeal and oropharyngeal swabs is uncomfortable for patients and may pose a risk to healthcare workers [3, 4]. In addition, the high demand for the materials and reagents needed to sample individuals and the requirement of professional technicians and trained skills to perform RT‒qPCR have resulted in inadequate community-based testing, which might contribute to thousands of preventable COVID-19-related deaths, especially in low- and middle-income countries [5, 6]. Thus, alternative specimens that can be conveniently and safely collected and compatible for low-cost point-of-care testing started attracting the attention of researchers in the first half of 2020.

Saliva has emerged as a promising noninvasive biofluid for the diagnosis of oral and systemic diseases not only for the abovementioned merits but also for the enrichment of various biomarkers, such as proteins, DNA, RNA, metabolites, and microorganisms [7–9]. Accumulating studies have shown that viral infections can be diagnosed using saliva instead of blood or urine [10]. A diverse set of viable viruses, including influenza virus A, HIV, and Ebola virus, have been isolated from saliva and oral swabs. The shedding of viral nucleic acid fragments and viral proteins and the secretion of host antibodies against viral proteins have been detected in saliva, indicating that saliva is an all-round specimen and could be used for the development of various testing techniques targeting different biomarkers [10, 11]. Of note, saliva is particularly suitable for the detection of respiratory viruses because it contains secretions coming down from the nasopharynx and coming up from the lung via the action of cilia lining the airway [12].

Pioneering studies on the detection of SARS-CoV-2 using saliva as a specimen were conducted in early 2020 [12–14]. After these studies, researchers have been increasing interested on saliva-based detection of SARS-CoV-2, and as a result, the number of publications in this research field increased from 0 to 1021 (data from 2023-01-24) during the past three years. These studies could briefly be classified into three main categories, and the listed references are examples. The first type is clinical studies investigating saliva as a potential route of SARS-CoV-2 transmission or a reliable diagnostic specimen by comparing SARS-CoV-2 detection between nasopharyngeal and saliva via established standard methods [14–16]. The second type is the development of novel saliva-based techniques for SARS-CoV-2 detection, including CRISPR/Cas13-based isothermal amplification assays, electrochemical immunoassays, and surface-enhanced Raman spectroscopy (SERS) immunoassays [17–19]. These methods are usually verified using clinical samples or saliva spiked with targeted molecules. The third type is reviews, including progress and perspective reviews, systematic reviews, and meta-analyses [20–23]. However, several scientific questions related to the basic knowledge landscapes in research on saliva-based detection of SARS-CoV-2 remain elusive. These questions include the following: To what extent has this research area developed? What are the past, current and future hotspots in this research area? What is the contribution and influence of countries, institutions and authors to this research area?

Bibliometric analysis is a novel systematic and scientific method used to explore and evaluate contributions to certain research areas, including contributions from countries and regions, institutions, authors, journals, and articles. Furthermore, bibliometric analysis is able to summarize hotspots and predict trends in certain research areas through visualization analysis of massive information [24–27]. CiteSpace is one of the most common software programs used for bibliometric and visualization analysis [25, 28, 29]. However, a bibliometric analysis of research on saliva-based detection of SARS-CoV-2 has not been performed. In this study, we utilized the WoS core collection (WoSCC) as a literature database and CiteSpace as visualization software and conducted a comprehensive bibliometric analysis of research on saliva-based detection of SARS-CoV-2 from 2020 to 2023 to explore its hotspots and trends. We hope that our study will provide a new perspective and foundation for future research on saliva-based detection of SARS-CoV-2, as well as other viral infections.

## Materials and methods

### Data source and retrieval strategies

**Fig. 1:**
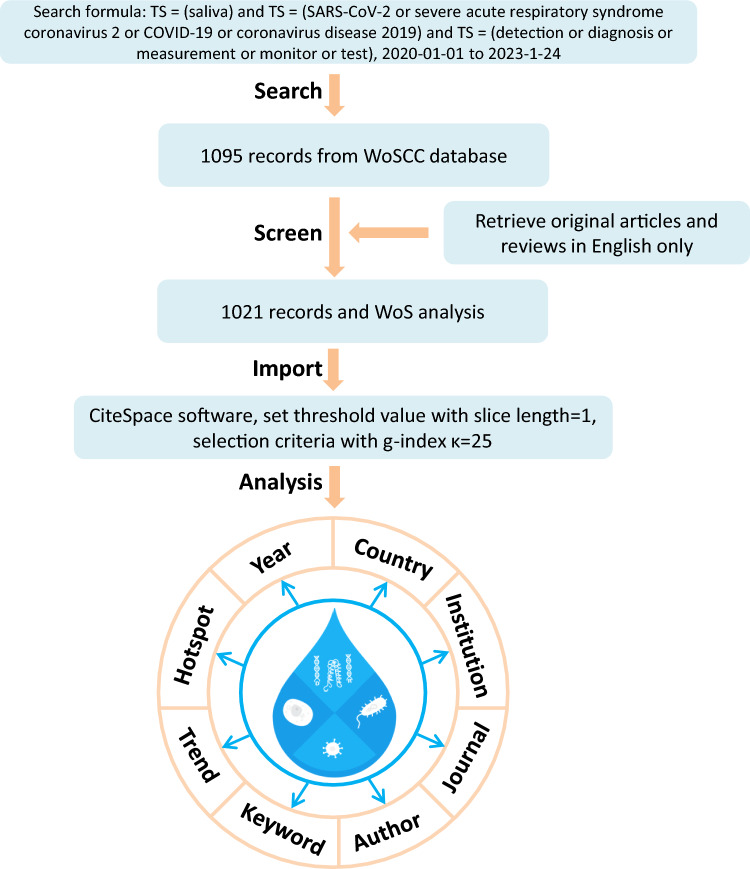
Flow chart of the literature search of the WoSCC, the screening process, and the importing of data into CiteSpace for analysis

WoSCC was used as the data source for the literature search. The search formula was TS = (saliva) and TS = (SARS-CoV-2 or severe acute respiratory syndrome coronavirus 2 or COVID-19 or coronavirus disease 2019) and TS = (detection or diagnosis or measurement or monitor or test). The time period was 2020-01-01 to 2023-01-24. A total of 1095 records were obtained. Of the various publication types, only original articles and reviews published in English were included in our study. After screening, 1021 valid publications were retrieved, analyzed by WoS, and imported to CiteSpace for visualization analysis (Fig. 1).

### Data analysis

CiteSpace (version 5.6 R4), a JAVA program-based software program for creating scientific knowledge maps, was used in this study to analyze and visualize the literature related to saliva-based detection of SARS-CoV-2. In the visual maps generated by CiteSpace, betweenness centrality measures the number of times a certain node lies on the shortest path between the other nodes. Nodes with high betweenness centrality usually connect various clusters and are recognized as key hubs [28].

The retrieved literature was analyzed by WoS for research field, journal, and citation report. After importing the data into CiteSpace, the time parameters were set from 2020 to 2023 with a time slice of 1 year, and the selection criteria were set with g-index *κ* = 25. The node types included in this study were country, institution, author, cited author, cited journal, and keyword. A keyword cooccurrence map was used to study the research hotspots over the years. A keyword clustering map was utilized to decipher the relevant content of a certain type of topic research. Keywords timeline viewer was used to obtain the development relationship between research hotspots. The modularity (*Q* value) and silhouette (*S* value) were used to evaluate the structure of the clustering network. A *Q* value > 0.3 indicates that the cluster structure is significant, and *S* value > 0.3, 0.5, or 0.7 indicates that the network is homogenous, reasonable, or highly credible, respectively [28, 30]. The map clipping methods were set to Pathfinder, Pruning sliced networks, and Pruning the merged network. Microsoft Excel 2007 was used to plot the annual trends of publications and citations.

## Results

### Overview of publications and citations on saliva-based detection of SARS-CoV-2

**Fig. 2:**
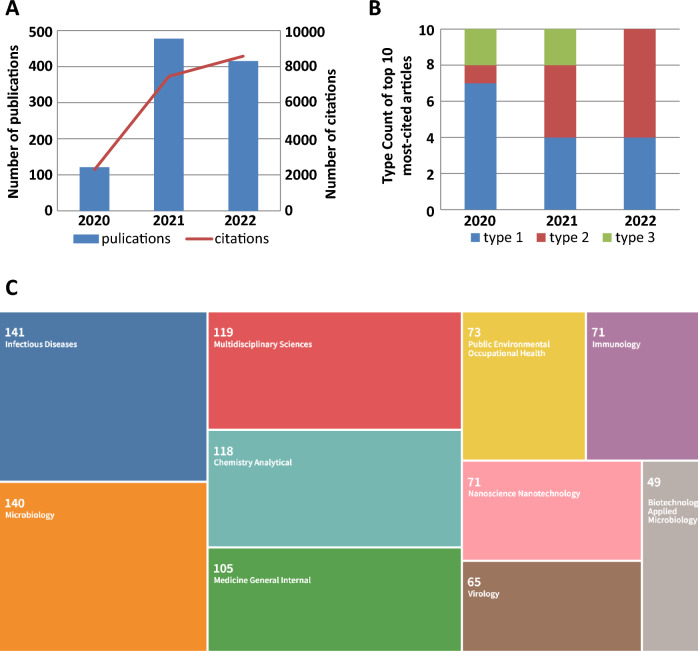
Analysis of annual publications and citations (**A**), composition of the top 10 most-cited articles published in 2020, 2021, and 2022 (**B**) and fields of the WoS categories associated with saliva-based detection of SARS-CoV-2 (**C**). The articles were classified into three main types: clinical studies (type 1), saliva-based new methodologies (type 2), and reviews (type 3)

Based on the literature search of the WoSCC database, 1021 articles related to saliva-based detection of SARS-CoV-2 were published in the past 3 years and included in our study for visualization analysis. The detailed retrieval strategy and analysis procedure are shown in Fig. 1. Citation reports from WoS showed that the number of publications in this field exhibited a sharp increase from 2020 to 2021 and a slight decrease from 2021 to 2022 (Fig. 2A). In contrast, the number of citations increased annually, indicating a growing influence of this research area. The overall number of citations of the retrieved articles was 18,632, and the average number of citations per article was 18.25. The H-index for all publications was 58.

Table 1 shows the top 10 most-cited articles: 9 of 10 were published in 2020, and 7 out of 10 were type 1 articles according to the three main types. Type 1 refers to clinical studies investigating saliva as a transmission route and diagnostic specimen, type 2 articles describe the development of new saliva-based methodologies for SARS-CoV-2 detection, and type 3 constitutes reviews. A comparison of the top 10 most-cited articles published in a single year revealed a gradual decrease in type 1 articles and a gradual increase in type 2 articles from 2020 to 2022 (Fig. 2B). This finding indicates that scientists initially concerned saliva as a transmission route and diagnostic specimen, but later exhibited more interest in new saliva-based methodologies.

**Table 1:** Top 10 most-cited articles in research on saliva-based detection of SARS-CoV-2

Rank	First author	Journal	Publication date	Total citations	Category	References
1	To KK	Lancet Infectious Diseases	MAY 2020	1935	Type 1	[13]
2	To KK	Clinical Infectious Diseases	AUG 2020	1075	Type 1	[12]
3	Zheng S	BMJ-British Medical Journal	APR 2020	853	Type 1	[31]
4	Gandhi RT	New England Journal of Medicine	OCT 2020	687	Type 3	[35]
5	Cheng VC	Journal of Infection	JUL 2020	419	Type 1	[32]
6	Azzi L	Journal of Infection	JUL 2020	374	Type 1	[14]
7	Huang N	Nature Medicine	MAY 2021	260	Type 1	[16]
8	Nagura-Ikeda M	Journal of Clinical Microbiology	SEP 2020	230	Type 1	[33]
9	Wu SY	Expert Review of Molecular Diagnostics	SEP 2020	223	Type 3	[36]
10	Torrente-Rodriguez RM	Matter	DEC 2020	205	Type 2	[34]

The articles were categorized into three main types: clinical studies (Type 1), saliva-based new methodologies (Type 2) and reviews (Type 3)

Because the research progress and trends could be reflected by the most-cited articles in some regards, we briefly summarize the top 10 most-cited articles (Table 1). To et al. and Zheng et al. performed cohort studies to evaluate the salivary viral loads at different stages of COVID-19 and found that the salivary viral load was highest during the first week after symptom onset [13, 31]. Cheng et al. showed that community-wide mask wearing contributes to the control of COVID-19, probably by blocking the emission of infected saliva [32]. In May 2021, Huang et al. confirmed that epithelial cells of the salivary glands and oral mucosa were infected by SARS-CoV-2 and that saliva sustained SARS-CoV-2 infection. Thus, saliva is a potential transmission route during the COVID-19 pandemic [16]. Clinical evaluation with saliva samples from COVID-19 patients was then conducted, and these studies concluded that saliva is a reliable specimen for detecting SARS-CoV-2 RNA and protein by RT‒qPCR and rapid antigen tests [12, 14, 33]. Of note, saliva is a perfect candidate for point-of-care testing (POCT), and various saliva-based POCT techniques have been developed to detect SARS-CoV-2. As early as Dec 2020, Torrente-Rodríguez et al. developed a multiplexed electrochemical graphene-based platform for the sensitive, rapid, and simultaneous detection of SARS-CoV-2 antigen, IgM and IgG antibodies and the inflammatory biomarker C-reactive protein (CRP), and this platform has been successfully used with saliva samples from COVID-19 patients [34]. Moreover, Gandhi et al. and Wu et al. reviewed the diagnostic methods of COVID-19, including the most recently validated specimen type, saliva [35, 36].

Saliva-based detection of SARS-CoV-2 covered multiple fields of WoS categories, including infectious diseases, microbiology, and multidisciplinary sciences (Fig. 2C). Thus, the studies on saliva-based detection of SARS-CoV-2 not only cover infectious diseases (141, 13.81%), microbiology (140, 13.71%), and virology (65, 6.37%) but also concern multidisciplinary sciences (119, 11.66%), analytical chemistry (118, 11.56%), nanoscience nanotechnology (71, 6.95%), and immunology (71, 6.95%). Indeed, the development of novel saliva-based techniques for SARS-CoV-2 detection using nanotechnology, multidisciplinary, immunological, and analytical methods is a major topic in this research area.

### Distribution of countries and institutions

**Fig. 3:**
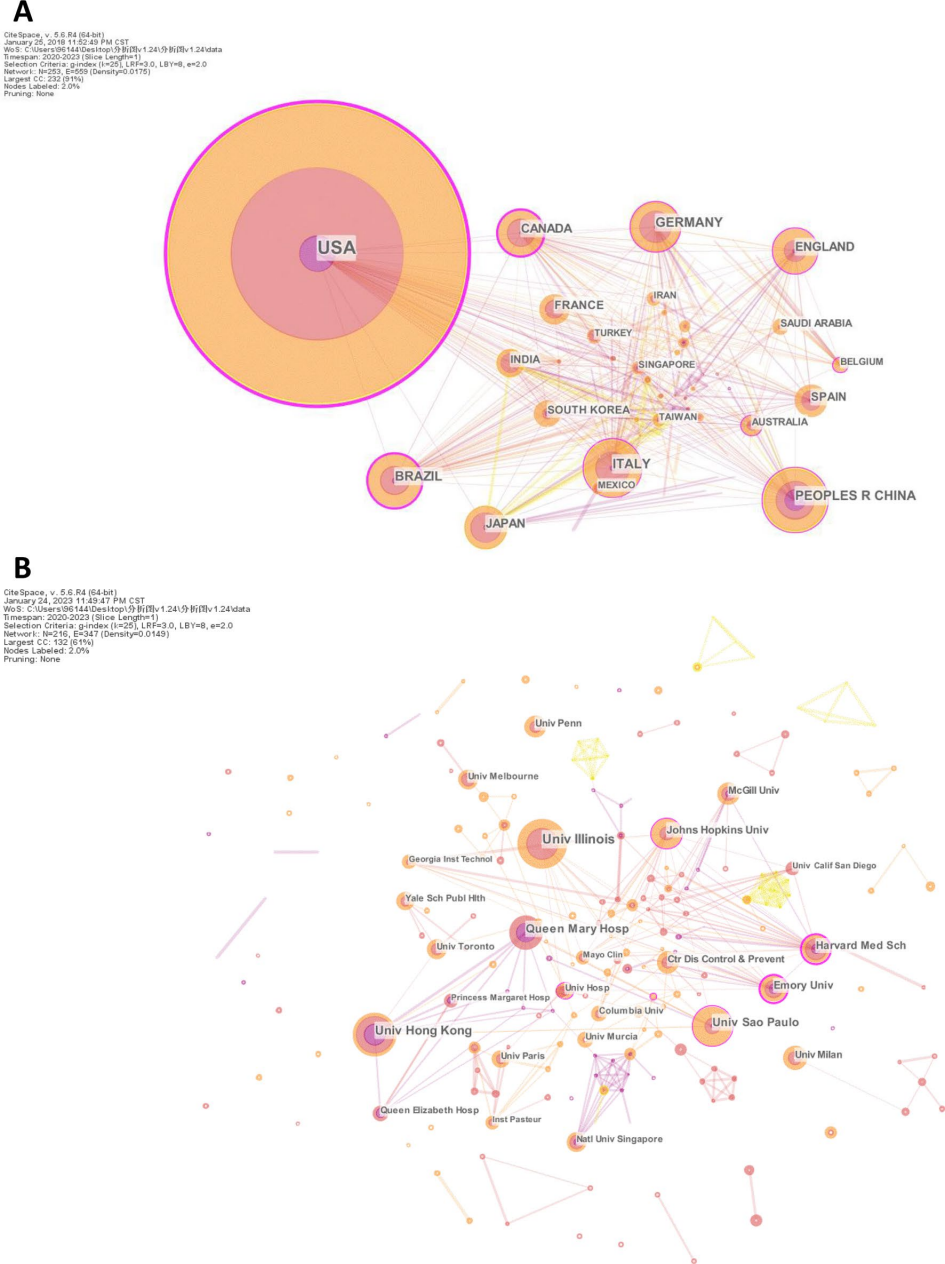
Visualization map of countries (**A**) and institutions (**B**) involved in research on saliva-based detection of SARS-CoV-2

Visualization analysis of the number of publications describing research performed in different countries and at different institutions would enable the identification of key countries and institutions that have a significant impact on certain research areas and their cooperative relationships. A total of 93 countries and regions and 2158 institutions participated in the publication of these articles related to saliva-based detection of SARS-CoV-2. The United States of America (USA) had the largest number of publications (322, 31.54%), and this number was markedly higher than that found for other countries and regions (Fig. 3A and Table 2). China, Italy, Brazil, and Germany ranked second to fifth in productivity. Seven out of the top 10 countries were distributed in North America and Europe, suggesting the dominant role of these continents in the research area. However, the ranking of betweenness centrality that reflects the influence of a node on the relationships in the network is slightly different from that of the publication number. The USA had the highest betweenness centrality (0.59), followed by Canada (0.39) and Brazil (0.37), indicating that these countries developed extensive international cooperation.

**Table 2:** Top 10 countries and institutions in terms of the number of publications on saliva-based detection of SARS-CoV-2

Rank	Country			Institution		
	Country	Count	Centrality	Institution	Count	Centrality
1	USA	322	0.59	Univ Illinois	25	0.09
2	PEOPLES R CHINA	71	0.15	Univ Hong Kong	22	0.06
3	ITALY	64	0.17	Univ Sao Paulo	20	0.11
4	BRAZIL	59	0.37	Queen Mary Hosp	17	0.03
5	GERMANY	56	0.17	Johns Hopkins Univ	15	0.1
6	CANADA	49	0.39	Harvard Med Sch	14	0.31
7	JAPAN	49	0.08	Emory Univ	13	0.31
8	ENGLAND	48	0.14	Univ Milan	12	0
9	SPAIN	36	0.04	Ctr Dis Control & Prevent	11	0.04
10	FRANCE	34	0.06	McGill Univ	11	0.01

A total of 11 institutions were responsible for more than 1% of publications. The University of Illinois published the highest number of articles (25, 2.45%), followed by the University of Hong Kong and University of Sao Paulo (Fig. 3B and Table 2). Half of the top 10 institutions with the highest number of publications are located in the USA, and this finding indicates that the USA is the country with the greatest research productivity on saliva-based detection of SARS-CoV-2. Although the University of Illinois ranked first in terms of the number of publications, the centrality metrics showed that Harvard Medical School (0.31) and Emory University (0.31) were the most influential institutions based on their comprehensive collaboration with other institutions.

### Analysis of authors and cited authors

**Fig. 4:**
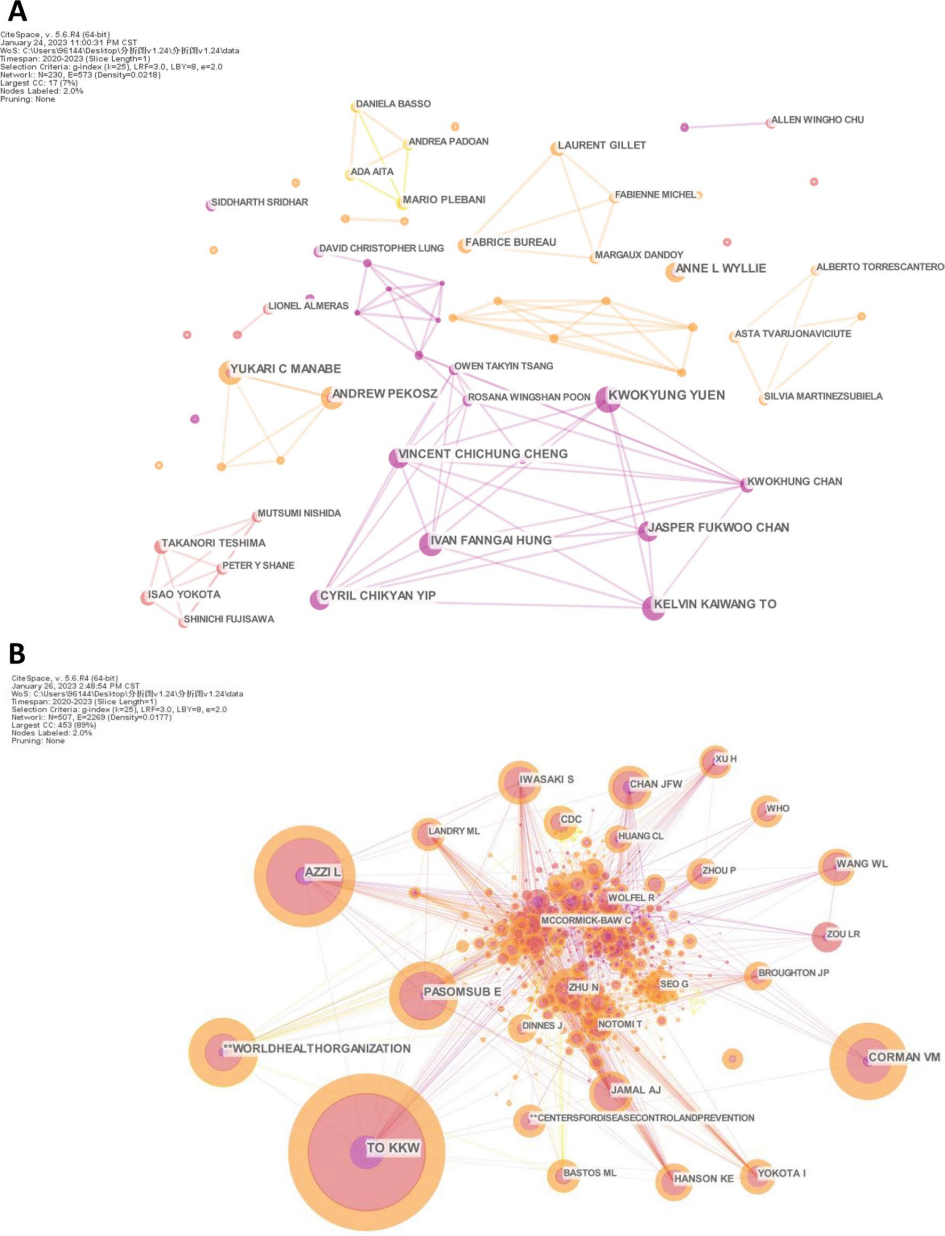
Visualization map of authors (**A**) and cited authors (**B**) involved in research on saliva-based detection of SARS-CoV-2

The authorship map had 230 nodes, 573 connections, and a network density of 0.0218. In total, 9566 authors contributed to this research area. The top 10 authors with the most publications and citations are listed in Table 3. Yuen KY from the University of Hong Kong published the highest number of articles, followed by Pekosz A and Manabe YC from Johns Hopkins University and Hung IF and To KK from the University of Hong Kong (Fig. 4A and Table 3). It is worth noting that most of the top 10 authors were from the same institutions or research teams and focused on clinical studies investigating saliva as a transmission route or diagnostic specimen. Surprisingly, their centralities were 0, indicating a lack of cooperation between these research teams in the context of the COVID-19 pandemic.

**Table 3:** Top 10 authors in terms of the number of publications and citations in research on saliva-based detection of SARS-CoV-2

Rank	Author’s publication volume		Author’s citation volume	
	Author	Count	Author	Count
1	Yuen KY	9	To KK	291
2	Pekosz A	8	Azzi L	189
3	Manabe YC	8	Corman VM	145
4	Hung AF	8	WHO	130
5	To KK	8	Pasomsub E	129
6	Wyllie AL	7	Zhu N	85
7	Yip CC	7	Jamal AJ	83
8	Cheng VC	7	Iwasaki S	83
9	Chan JF	7	Chan JF	82
10	Chan KH	5	Wang WL	72

An analysis of cited authors indicates the degree of recognition by other researchers and their influence within the network. The most-cited author was To KK, with a citation count of 291 (Fig. 4B and Table 3). Prof. To KK and his team have been devoted to improving clinical practice and guiding real-time public health responses to respiratory tract infections, including the development and evaluation of novel diagnostic tests and elucidation of the pathogenesis, viral kinetics, host response, and long-term immunity of COVID-19 [12, 13, 37, 38]. Azzi L from University of Insubria, Corman VM from Charité-Universitätsmedizin Berlin, World Health Organization (WHO) and Pasomsub E from Mahidol University ranked second to fifth, each with a citation count greater than 100. Interestingly, To KK and Chan JF were the only two scientists found among the top 10 authors and the top 10 cited authors, suggesting that they are the most productive and influential scientists in the research area.

### Analysis of journals and cited journals

**Fig. 5:**
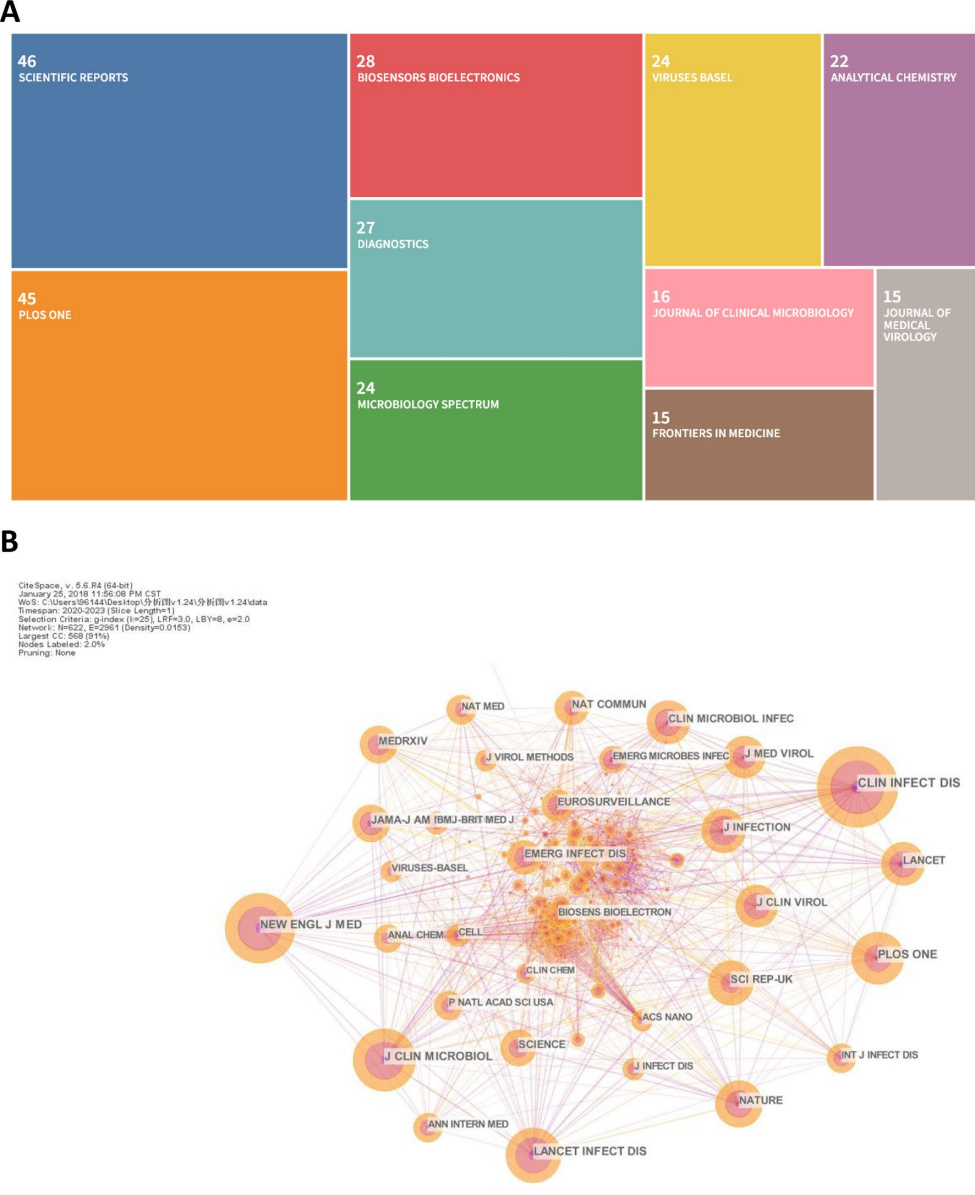
Visualization analysis of journals (**A**) and cited journals (**B**) related to saliva-based detection of SARS-CoV-2

In total, 1021 articles were published in 371 journals. Scientific Reports (46, 4.51%) and PLoS One (45, 4.41%), two open access scientific journals covering almost all areas of the natural sciences, were ranked first and second in terms of publication number (Fig. 5A and Table 4). The list of journals that contributed more than 20 publications included Biosensors & Bioelectronics, Diagnostics, Microbiology Spectrum, Viruses-BASEL, and Analytical Chemistry. It is expected that Diagnostics, Microbiology Spectrum, and Viruses-BASEL because the focus of these journals is closely related to SARS-CoV-2. The presence of Biosensors & Bioelectronics and Analytical Chemistry in this list indicated that scientists in the field of analytical chemistry have played an active role in research on saliva-based detection of SARS-CoV-2.

**Table 4:** Top 10 journals in terms of the number of publications and citations in research on saliva-based detection of SARS-CoV-2

Rank	Journal’s publication volume		Journal’s citation volume	
	Journal	Count	Journal	Count
1	Scientific Reports	46	Clinical Infectious Diseases	509
2	PLoS One	45	New England Journal of Medicine	443
3	Biosensors Bioelectronics	28	Journal of Clinical Microbiology	394
4	Diagnostics	27	Lancet Infectious Diseases	351
5	Microbiology Spectrum	24	PLoS One	329
6	Viruses-Basel	24	Nature	298
7	Analytical Chemistry	22	Scientific Reports	286
8	Journal of Clinical Microbiology	16	Journal of Infection	284
9	Frontiers in Medicine	15	Lancet	281
10	Journal of Medical Virology	15	Clinical Microbiology and Infection	277

An analysis of cited journals is able to disclose the authority and influence of the journals in the community of a specific research area. Figure 5B and Table 4 show the highly cited journals. The most-cited journal is Clinical Infectious Diseases (509), followed by New England Journal of Medicine (443), Journal of Clinical Microbiology (394), and Lancet Infectious Diseases (351). Thus, these journals exhibit comprehensive authority and influence in research on saliva-based detection of SARS-CoV-2. The findings also indicate that scientists in the fields of infection, medicine, and clinical microbiology are committed to addressing the scientific questions related to saliva-based detection of SARS-CoV-2. Among the top 10 journals with the most publications, the following three journals were also included in the list of top 10 most-cited journals: Journal of Clinical Microbiology, PLoS One, and Scientific Reports. Of note, the high citation number of these journals might be attributed or partially attributed to their high publication number. We also noticed that the journal impact factor (IF) of the top 10 journals in terms of citations (average IF of 61.31) was generally higher than that of the top 10 journals in terms of publications (average IF of 8.56).

### Analysis of keyword cooccurrence

**Fig. 6:**
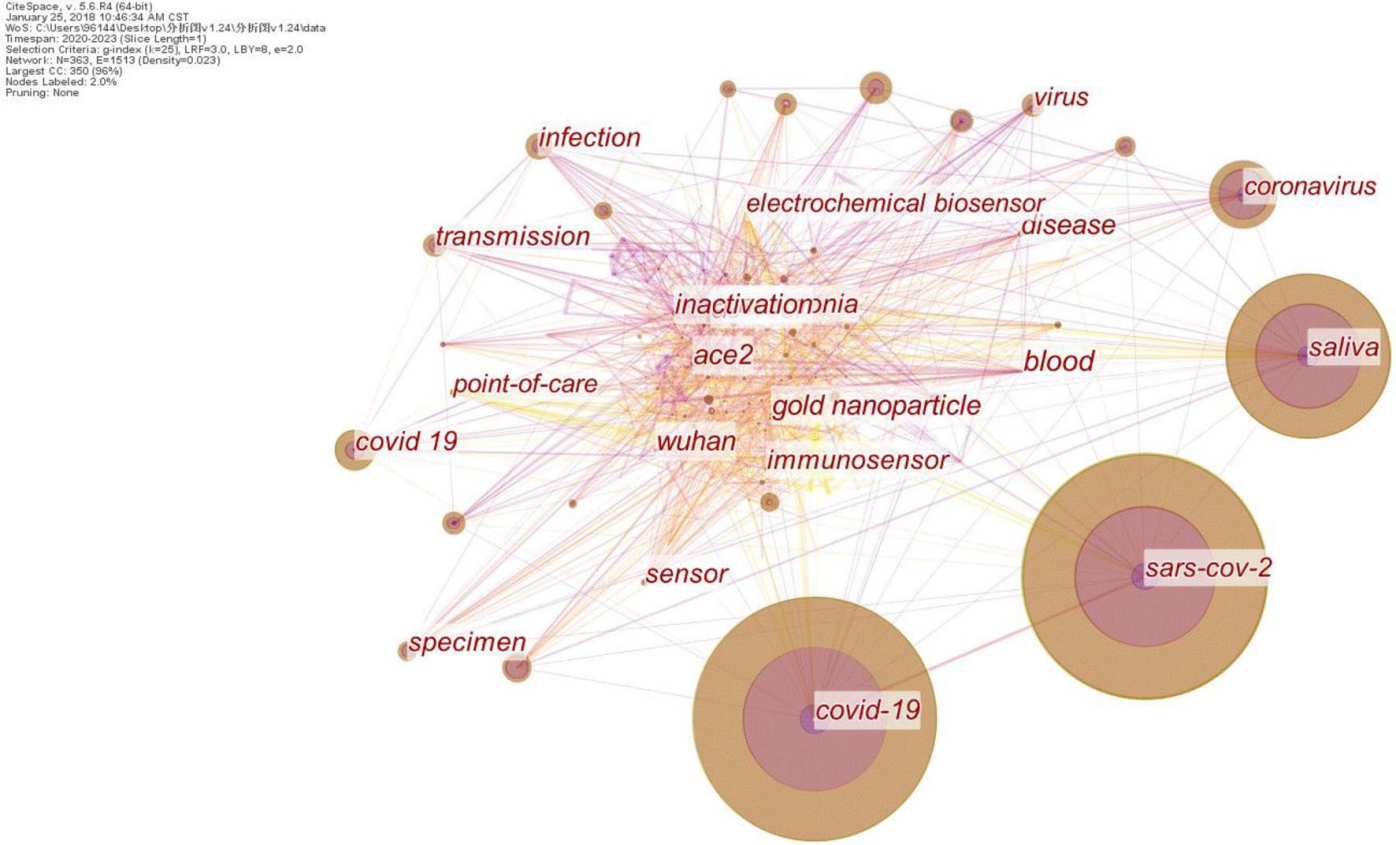
Visualization map of keyword cooccurrence related to saliva-based detection of SARS-CoV-2

Because keywords are highlighted summaries of the literature topic and content, the frequency and centrality of keywords generally reflect the intensity of a certain topic that researchers may be concerned with or aim to address in the research field [39]. The keyword cooccurrence map showed that SARS-CoV-2, COVID-19, saliva, and coronavirus were the most frequent and established extensive links to other keywords (Fig. 6). After excluding the same keywords used for the literature search, the top 20 keywords in terms of frequency were listed in Table 5. Interestingly, the top 20 keywords could fit into 3 categories. Keywords including infection, transmission, viral load, and aerosol were related to the oral cavity as an infection site and saliva as a transmission route (category 1a). Keywords including nasopharyngeal swab, RT‒PCR, PCR, specimen, RT‒qPCR, sensitivity, and swab were related to saliva as a specimen for SARS-CoV-2 detection (category 1b). The rest of top 20 keywords were antibody, biosensor, assay, mediated isothermal amplification, protein, and biomarker, which were related to development of novel saliva-based methodologies (category 2). Thus, analysis of keyword cooccurrence revealed that the major research themes were verification of saliva as transmission route and diagnostic specimen, and development of novel saliva-based methodologies.

**Table 5:** Top 20 keywords for research on saliva-based detection of SARS-CoV-2 (excluding keywords used for the literature search)

Rank	Keyword	Count	Centrality	Category
1	Nasopharyngeal swab	57	0.03	Type 1b
2	Infection	54	0.04	Type 1a
3	Transmission	46	0.06	Type 1a
4	Antibody	44	0.04	Type 2
5	RT‒PCR	44	0.04	Type 1b
6	PCR	41	0.02	Type 1b
7	Specimen	38	0.06	Type 1b
8	Biosensor	37	0.03	Type 2
9	Assay	32	0.01	Type 2
10	Viral load	25	0.04	Type 1a
11	RT‒qPCR	24	0.03	Type 1b
12	Sensitivity	24	0.01	Type 1b
13	Aerosol	24	0.07	Type 1a
14	Children	22	0.03	Type 1b
15	Mediated isothermal amplification	22	0.01	Type 2
16	Swab	20	0.03	Type 1b
17	Protein	18	0.03	Type 2
18	Surveillance	18	0.02	Type 1a
19	Biomarker	17	0.04	Type 2
20	Dentistry	17	0.03	Type 1a

The keywords were categorized into three main types: clinical studies related to the oral cavity as the infection site and saliva as the transmission route (Type 1a), clinical studies related to saliva as the specimen (Type 1b) and new saliva-based methodologies (Type 2)

### Hotspots and trends of saliva-based detection of SARS-CoV-2

**Fig. 7:**
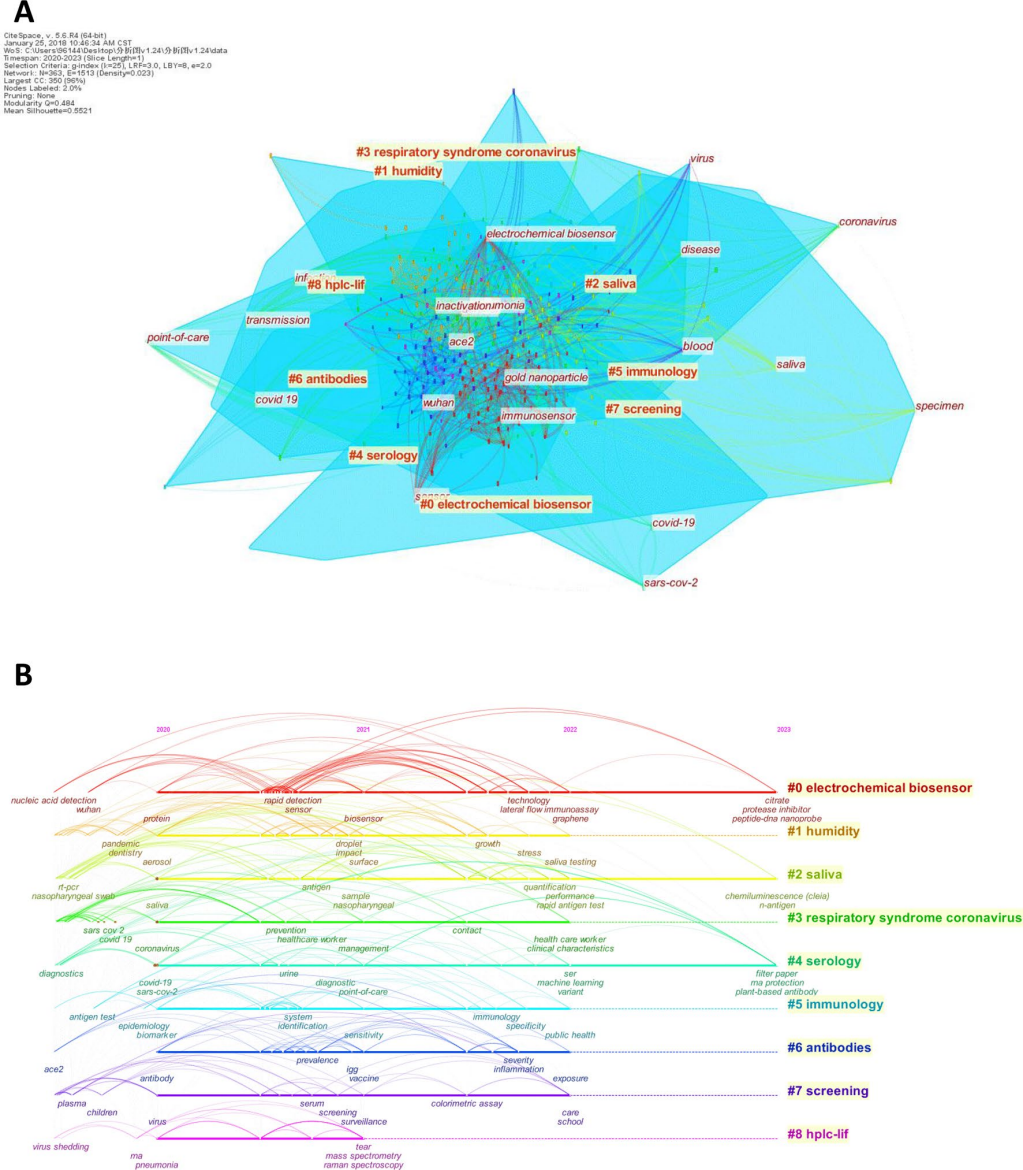
Visualization map of keyword clusters (**A**) and timelines (**B**) related to saliva-based detection of SARS-CoV-2

Based on the keyword cooccurrence analysis, keyword clusters can be formed from the most frequently used and related keywords. The keyword clustering map summarizes the research hotspots and elucidates the basic knowledge structure of certain research areas [40]. A keyword clustering map with 363 nodes, 1513 connections, a density of 0.023, a modularity (*Q* value) of 0.484 (> 0.3), and a silhouette (*S* value) of 0.5521 (> 0.5) was then obtained (Fig. 7A). Thus, the cluster structure is considered significant and reasonable [28, 30].

Nine keyword clusters were generated from the most frequently used keywords (Fig. 7A). An analysis of these clusters revealed that cluster #0 “electrochemical biosensors” mainly focused on the development of novel electrochemical immunosensors and aptasensors for rapid antigen tests [34, 41]. Cluster #1 “humidity” focused on wastewater surveillance and risk assessment for the community-wide monitoring of COVID-19 disease incidence [42, 43]. Cluster #2 “saliva” was related to detection of SARS-CoV-2 using saliva as a specimen [14, 33]. Cluster #3 “respiratory syndrome coronavirus” was associated with infection prevention and medical treatment, and cluster #4 “serology” was related to nanoparticle-based immunosensors with the application of differential pulse voltammetry for SARS-CoV-2 detection [18, 44]. Cluster #5 “immunology” mainly focused on rapid testing using immunoassays, molecular techniques and biotechnology. Cluster #6 “antibodies” was associated with mucosal and humoral immunity and oral inflammatory diseases. Cluster #7 “screening” mainly focused on biomarkers used for screening respiratory diseases, and cluster #8 “HPLC-LIF” was related to the rapid detection of SARS-CoV-2 by high-performance liquid chromatography-laser induced fluorescence (HPLC-LIF) [45]. Overall, 6 out of 9 clusters (#0, #2, #4, #5, #7, #8) were closely associated with saliva-based detection of SARS-CoV-2 using various multidisciplinary technologies.

A timeline view of keywords can track the explosion and decline of a certain topic over time. Figure 7B shows the keyword timeline for this research area. Although research on saliva-based detection of SARS-CoV-2 has been conducted for only three years, some trends have already been observed. Most of the clusters, including clusters #0, #2, and #4, remained as hotspots from 2020 to 2022. However, cluster #8 suddenly declined in 2022. The keywords “saliva,” “epidemiology,” and “biomarker” first appeared in 2020 and are still active as hotspots, indicating the broad application of salivary biomarkers in diagnosis of epidemic diseases. “Biosensor” and “point-of-care” have emerged as hotspots since 2021, coincident with the appearance of various biosensors for SARS-CoV-2 detection, including electrochemical immunoassay-based biosensors, SERS-based biosensors, and field-effect transistor (FET)-based biosensors [18, 19, 46]. The employment of “machine learning” technology in SARS-CoV-2 detection became a hotspot in 2022 and might continue to play an active role in aiding medical diagnosis and therapeutic intervention [47–49].

Research themes usually go through three stages: incubation, development, and decline. While keyword cooccurrence pointed out the major research themes, i.e., verification of saliva as transmission route and diagnostic specimen, and development of novel saliva-based methodologies, keyword timeline could organize them into different stages. We found that two stages were formed since the beginning of research on saliva-based detection of SARS-CoV-2. The first stage is 2020 to 2021, when the major research theme was viral transmission via saliva and verification of saliva as a reliable specimen. The second stage is 2021 to the present, when the major research theme was switched to the development of saliva-based multidisciplinary biosensors for SARS-CoV-2 detection. This finding was consistent with the results from the analysis of the annual trends of the most-cited articles (Fig. 2B).

## Discussion

Saliva plays a versatile role in human health, including its therapeutic effect and diagnostic implication. According to the theories of traditional Chinese medicine recorded in the “Compendium of Materia Medica,” saliva is able to irrigate the viscera, moisten the body, prevent diseases, and benefit longevity. Currently, the oral cavity and saliva have been widely considered not in isolation but as a component integrated with systemic physiology that plays important roles in maintaining systemic health and is reflective of systemic disease [50]. The reflective or diagnostic role of saliva has increased the attention of researches toward saliva-based diagnosis of oral diseases and systemic diseases, including viral infections [7, 8, 10]. Saliva exhibits comparable sensitivity and specificity to nasopharyngeal specimens in the detection of respiratory viruses, including coronaviruses [51, 52]. It is no surprise that saliva was used as a noninvasive specimen for SARS-CoV-2 detection immediately after the outbreak of the COVID-19 pandemic.

### The dual roles of saliva

As early as 2020-02-12 (online published date), pioneering work conducted by To et al. detected SARS-CoV-2 RNA in the saliva of COVID-19 patients, with a concordance rate of 91.7% (11/12) with nasopharyngeal specimens [12]. Since then, numerous efforts have been devoted to seeking answers to the following three questions. The first question is what biomarker types could be provided by saliva for the detection of SARS-CoV-2 infection. Initial studies focused on testing SARS-CoV-2 RNA in saliva [13, 14]. More recently, SARS-CoV-2 structural proteins (mainly S protein and N protein) and IgG, IgA and IgM antibodies against viral proteins were detected in saliva from COVID-19 patients [33, 34, 53], supporting the notion that saliva is a versatile specimen for detecting SARS-CoV-2 infection.

The second question is the extent to which saliva could be trusted to serve as a clinical specimen for detecting SARS-CoV-2 infection. Most studies were carried out in 2020 to compare the RT‒qPCR results between saliva and nasopharyngeal swabs from COVID-19 patients and healthy individuals. Azzi et al. found that all salivary samples of 25 COVID-19 patients tested positive for SARS-CoV-2 RNA, leading to the conclusion that saliva is a reliable tool for detecting SARS-CoV-2 [14]. Pasomsub et al. revealed that the RT‒PCR results reached 97.5% agreement between the two specimens: saliva and nasopharyngeal swab [54]. Butler-Laporte et al. included 15 independent studies in a meta-analysis and found that saliva and nasopharyngeal swabs shared similar sensitivity (83.2% *vs.* 84.8%) and specificity (99.2% *vs.* 99.8%) in detecting SARS-CoV-2 RNA [55]. Moreover, discordance has been found between saliva and nasopharyngeal swabs and among different studies. For some patients, SARS-CoV-2 RNA has been detected in saliva but not in nasopharyngeal swabs, which challenges the classification of nasopharyngeal swabs as the gold standard specimen [14, 21]. Hospitals accordingly changed the discharge policy for recovered COVID-19 patients to negative testing results with two pharyngeal swabs and one salivary swab [14]. Positive saliva and negative nasopharyngeal aspirate have also been observed in the detection of other respiratory viruses [51]. In addition, high between-person variability and false negative rates have been obtained with minimally processed saliva [56, 57], highlighting the need to optimize collection and processing before saliva could be applied as a standardized specimen for nucleic acid testing and large-scale screening of viral infections.

The third question is the mechanism underlying the existence of SARS-CoV-2 in saliva. Huang et al. and Song et al. showed that the SARS-CoV-2 receptors ACE2 and TMPRSS are enriched in epithelial cells of salivary glands and oral mucosa, which are shed into saliva [16, 58]. Salivary fractions from COVID-19 patients have been found to transmit SARS-CoV-2. Thus, saliva is a double-edged sword in combating COVID-19. On the one hand, saliva plays a “friend” role as a noninvasive and reliable specimen for the detection of SARS-CoV-2, and on the other hand, saliva also plays a “foe” role as an important transmission route of SARS-CoV-2 [59].

### Saliva-based novel point-of-care biosensor for SARS-CoV-2 detection

After the abovementioned questions were addressed, specifically after that saliva was verified as a reliable specimen for SARS-CoV-2 detection, scientists with various expertise then started to develop saliva-based or saliva-compatible POCT techniques for the rapid, sensitive, reliable, inexpensive, and scalable detection of SARS-CoV-2, including SERS, FET, electrochemical immunosensors and aptasensors [20, 60].

Although detecting any of SARS-CoV-2 RNA, antigens and antibodies in saliva could determine the infection status, most of saliva-based point-of-care biosensors aim to SARS-CoV-2 antigens (S protein, N protein, 3CL protease, and etc.) for rapid antigen tests. In Dec 2020, RapidPlex, a multiplexed electrochemical graphene-based platform, was developed for the rapid and sensitive detection of SARS-CoV-2 antigen, IgM and IgG antibodies and CRP [34]. RapidPlex successfully detected SARS-CoV-2 antigen and antibody in the saliva of COVID-19 patients. In July 2021, Torres et al. designed a handheld and highly sensitive electrochemical impedance spectroscopy (EIS) biosensor modified with ACE2 for the rapid detection of SARS-CoV-2 S protein [61]. The sensitivity and specificity of the EIS biosensor for saliva samples are 100% and 86.5%, respectively. Furthermore, the EIS biosensor exhibits no cross-reactivity with other respiratory viruses. In October 2022, Borberg et al. developed a carbon paper electrode (CPE)-based electrochemical biosensor that could detect active SARS-CoV-2 infection based on the enzymatic activity of viral 3CL protease [62]. Of note, the CPE biosensor detected viral infection within 1 min directly from unprocessed saliva samples.

### Findings of bibliometric analysis

By far, fast and great advances have been made in the research field of saliva-based detection of SARS-CoV-2. However, no bibliometric analysis has been performed to summarize the research progress, hotspots and trends in this area. Here, we conducted a bibliometric analysis of saliva-based detection of SARS-CoV-2. An analysis of countries and institutions revealed that the USA held a notable lead in this research area in regards to both contribution and influence (Fig. 3 and Table 2). China ranked 2nd in publication number but 8th in centrality, indicating disparities in its global collaboration compared with developed countries. The University of Illinois and the University of Hong Kong ranked first and second in terms of publication number, whereas Harvard Medical School and Emory University ranked first and second in centrality, consistent with the leader position of the USA. This finding also indicates that the University of Hong Kong has played an important role in this research area. Indeed, Yuen KY and To KK, the most productive and influential authors revealed by the analyses of published authors and cited authors, respectively, are both from the University of Hong Kong (Fig. 4 and Table 3). Interestingly, the journal analysis showed that Biosensors & Bioelectronics and Analytical Chemistry were among the top 10 journals with the most publications (Fig. 5 and Table 4). These findings demonstrate that scientists in the field of analytical chemistry have actively focused on and participated in saliva-based detection of SARS-CoV-2.

Bibliometric analysis usually takes advantage of timeline view of keywords to highlight the explosion and decline of a certain topic over time. We found that keywords “nasopharyngeal swabs,” “ACE2,” and “virus shedding” emerged as hotspots in 2020, coincident with initial efforts in verification of saliva as a diagnostic specimen and transmission route. The noninvasive and convenient collection of saliva makes saliva a perfect candidate for POCT, which is also reflected by “biosensors” and “point-of-care” as research hotspots in 2021 (Fig. 7B). Although graphene and machine learning were first integrated into biosensor of SARS-CoV-2 as early as in 2020 and 2021 [34, 63], “graphene” and “machine learning” became hotspots until 2022, indicating a gradual adoption of the initial innovation. “N antigen” emerged as a hotspot in 2023. As N protein is the most abundant protein of SARS-CoV-2 and is less prone to undergo mutation compared to S protein during viral transmission, future biosensors might widely utilize N protein as salivary biomarker.

### Perspectives on saliva-based viral detection

What are the future directions in the field of saliva-based detection of SARS-CoV-2 in the current transition from COVID-19 epidemic to endemic? Here, we list three points for discussion. First, the saliva collection and processing methods have varied among different studies, which might directly influence the testing results. Although several studies have shown that SARS-CoV-2 RNA could be detected in unprocessed saliva [64, 65], other studies found a high false negative rate and between-person variability using minimally processed saliva [56, 57]. In addition, the types of saliva (unstimulated, stimulated, unforced saliva, and forced deep throat saliva), the part of saliva (cell or cell-free), and behaviors before testing (eating and mouth washing) also interfere with SARS-CoV-2 detection [66–68]. Therefore, the methods of saliva sampling, processing, storing, and assaying need to be optimized and validated before being introduced as a standardized procedure in clinical applications.

Second, SARS-CoV-2 is not the only virus present in saliva. What other viruses could be found in saliva? Answering this question will not only explore saliva-based, noninvasive, and convenient detection of these viruses but also be helpful for the control of viral diseases due to the awareness of saliva as a potential transmission route.

Third but not least, many scientists with diverse expertise have relocated their resources to develop POCT techniques for SARS-CoV-2 detection. The impact of these efforts, however, will not diminish along with the decreased or ceased demand for SARS-CoV-2 detection. The strategies deployed and experiences learned from SARS-CoV-2 detection would shine the way to the development of POCT techniques for detecting other viruses, which is another interesting future direction. Overall, research on saliva-based detection of SARS-CoV-2 will significantly promote the development of diverse research fields, especially saliva-based diagnostics and biosensors for viral detection.

## Conclusion

In this study, we employed WoSCC as a literature database and CiteSpace as visualization software and conducted a bibliometric analysis of research on saliva-based detection of SARS-CoV-2 in terms of publications, citations, countries and regions, institutions, authors, cited authors, journals, cited journals, keyword cooccurrence, clusters and timelines. The research in this area initially focused on saliva as a diagnostic specimen and transmission route and switched to saliva-based multidisciplinary biosensors and POCT techniques for the rapid, sensitive, inexpensive, and scalable detection of SARS-CoV-2. Overall, saliva is a reliable specimen for the detection of viral RNA, antigens, and antibodies. However, a standardized procedure for saliva sampling and processing is needed, especially for the detection of viral RNA. Research on saliva-based detection of SARS-CoV-2 will promote the development of saliva-based diagnostics and biosensors for viral detection.

## Supplementary Information

Below is the link to the electronic supplementary material.

Supplementary file1 (XLSX 25 KB)

## Data Availability

The datasets presented in this study can be found in online repositories. The names of the repositories/repositories can be found in the article/supplementary material.
